# Capillary leak syndrome following cesarean section in a patient with severe preeclampsia: a case report

**DOI:** 10.3389/fmed.2025.1540957

**Published:** 2025-04-29

**Authors:** Haijuan Jin, Huimin Li, Junjun Cheng, Wenjuan Huang, Da Lin

**Affiliations:** ^1^Department of Obstetrics and Gynecology, Hangzhou Linping Hospital of Traditional Chinese Medicine, Hangzhou, China; ^2^Department of General Surgery, Hangzhou TCM Hospital Affiliated to Zhejiang Chinese Medical University, Hangzhou, China

**Keywords:** cesarean section, postpartum, capillary leak syndrome, severe preeclampsia, case report

## Abstract

**Background:**

Capillary leak syndrome (CLS) is a rare, often idiopathic condition, typically characterized by edema, hypotension, hypovolemic shock, and hypoalbuminemia. The progression of CLS is rapid, with a complex clinical course. If left undiagnosed or untreated, CLS can cause multiorgan failure and significantly increase the risk of mortality. Although CLS is generally associated with conditions such as infections, trauma, or autoimmune disorders, the occurrence of the condition following a cesarean section in patients with severe preeclampsia is exceedingly uncommon.

**Case description:**

A 37-year-old pregnant woman at 37 weeks of gestation underwent a cesarean section due to severe preeclampsia. Postoperatively, the patient developed sudden hypoxemia, massive ascites, oliguria, hypotension, and hypoalbuminemia. Following prompt identification and diagnosis, treatment was initiated with blood pressure management, magnesium sulfate to alleviate spasms, and supplementation with hydroxyethyl starch, albumin, and crystalloids. Additionally, corticosteroids were administered to improve capillary permeability. The condition was rapidly managed, with subsequent follow-up revealing no recurrence of similar issues.

**Conclusion:**

To the best of our knowledge, this is the first documented case of CLS occurring following a cesarean delivery in a patient with severe preeclampsia. The successful management of this patient offers valuable insights into early diagnosis, prompt treatment, and the potential to improve the prognosis of similar cases.

## Introduction

Clarkson first introduced the concept of capillary leak syndrome (CLS), also known as vascular leak syndrome, in 1960, with its idiopathic form commonly referred to as “Clarkson’s disease” (1). CLS is an acute, reversible condition characterized by increased vascular permeability, resulting in rapid leakage of plasma proteins and fluid into the interstitial spaces. This condition often leads to progressive edema, hypoalbuminemia, hypotension, decreased central venous pressure, and reduced hemoconcentration. In severe cases, CLS can cause multiorgan failure (2, 3). Although CLS is rare, its occurrence in pregnant women is even more uncommon. Abramov reported a case of postpartum CLS associated with the rupture of a benign ovarian cystic teratoma (4). Kanda also reported a case of postpartum CLS in an obstetric patient, where rapid disease progression necessitated transfer to the intensive care unit for emergency treatment (5). The disease progresses rapidly, carries a high mortality rate, and presents with non-specific symptoms, making diagnosis challenging. Misdiagnosis or delayed diagnosis can have severe consequences, with studies reporting acute CLS mortality rates as high as 20–30% (6). In this report, we present a previously undocumented case of CLS occurring after cesarean delivery in a patient with severe preeclampsia, which, to our knowledge, has not been reported in the literature.

## Case description

**Figure 1:**
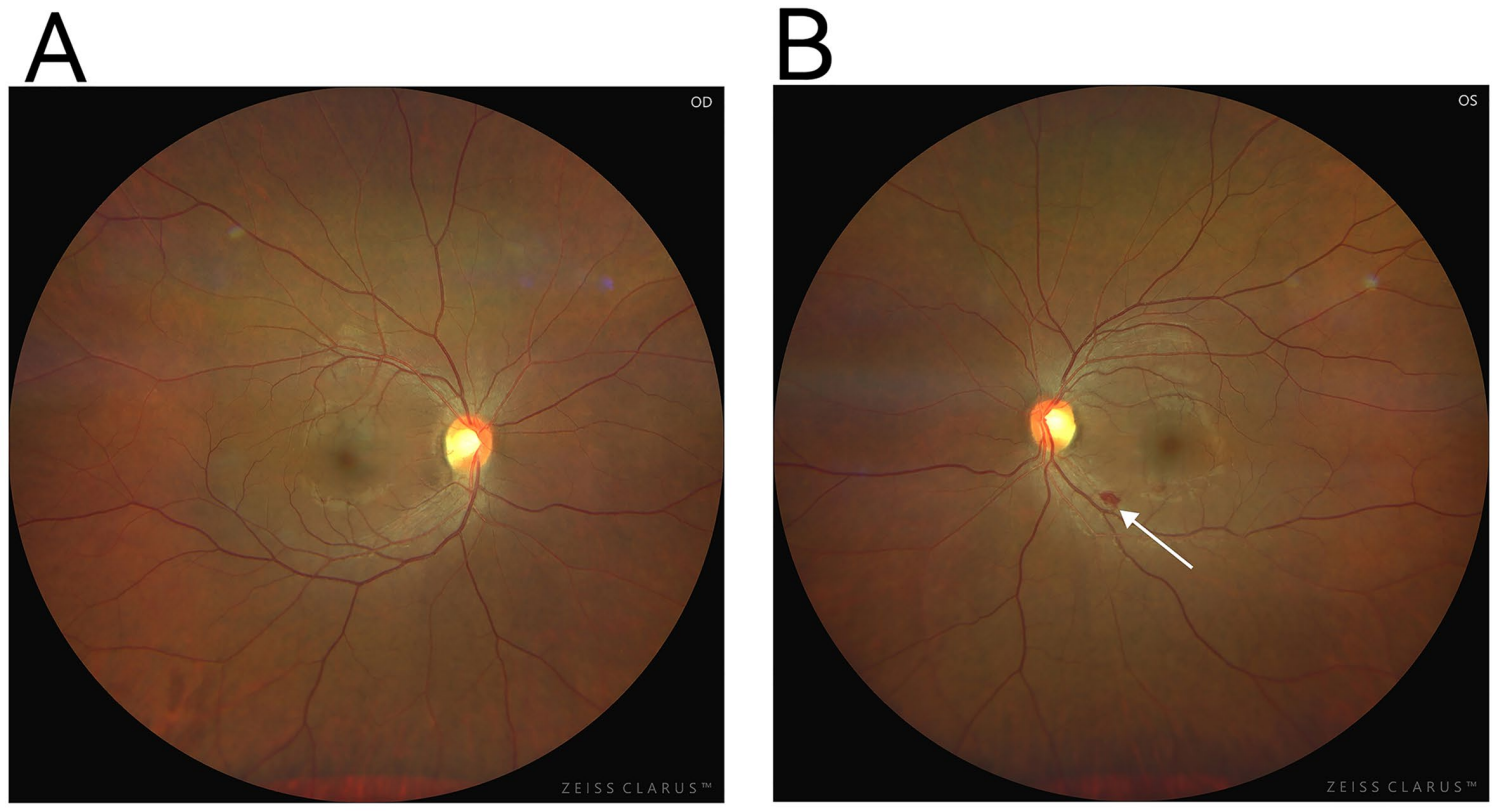
**(A)** Is the right fundus, **(B)** is the left fundus, and the arrow points to a patchy hemorrhagic lesion next to the large vessel below the macula in the posterior pole of the left eye.

**Figure 2:**
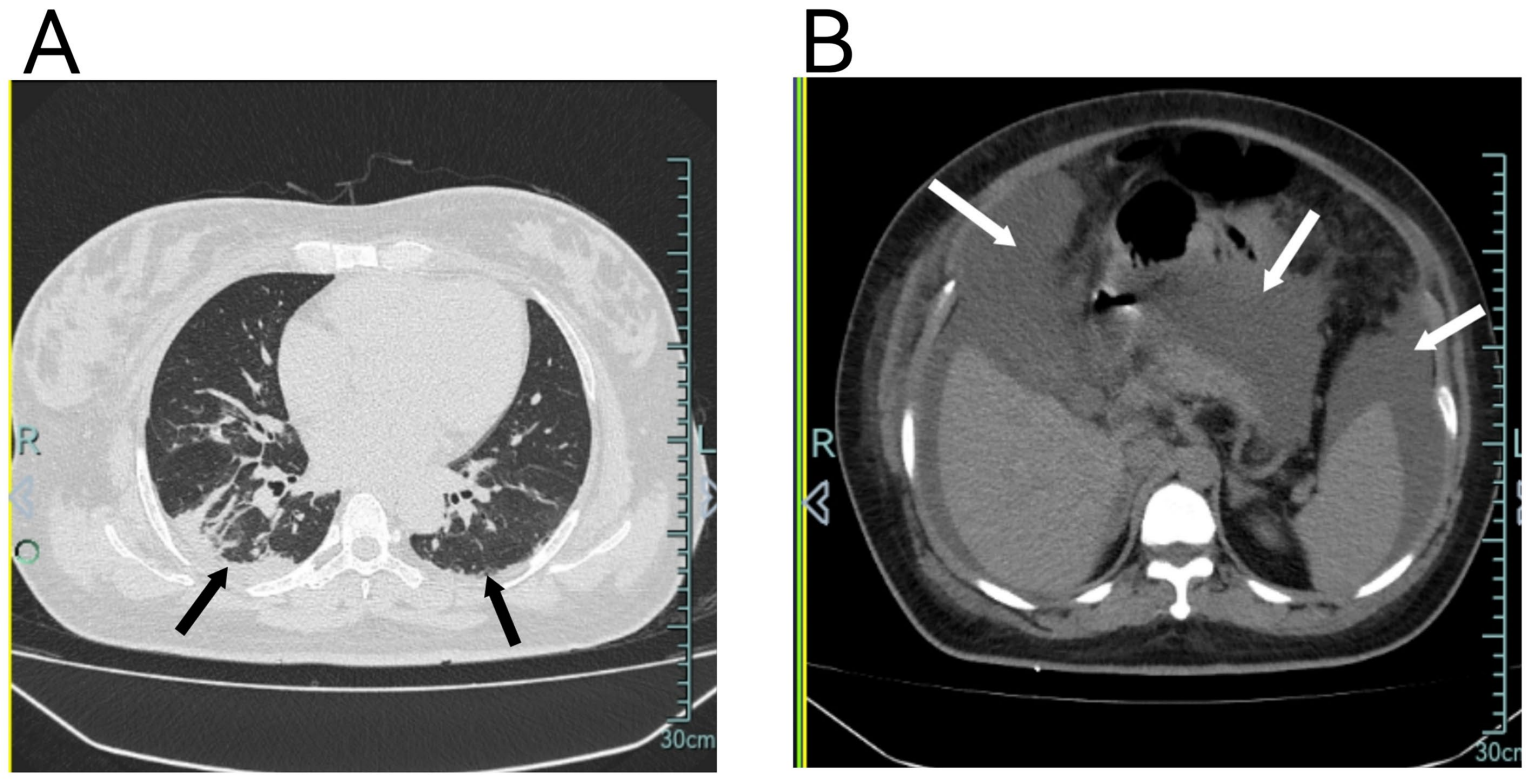
**(A)** Chest CT shows diffuse pulmonary exudates in both lungs, partial atelectasis in the right lower lobe, and minimal pleural effusion bilaterally. **(B)** Chest CT also demonstrates significant abdominal fluid accumulation.

A 37-year-old primigravida at 37 weeks and 2 days of gestation, with prior regular prenatal checkups and no significant medical history, was diagnosed with gestational diabetes during pregnancy. Additionally, no other known risk factors were observed. The patient presented with a complaint of bilateral lower extremity edema lasting for 2 weeks, along with recent difficulties in managing her blood pressure. Outpatient dynamic blood pressure monitoring revealed a 24-h average of 161/111 mmHg, and urinalysis confirmed proteinuria (+), prompting her admission for further evaluation and management. Upon admission, laboratory tests and ultrasound imaging demonstrated no significant abnormalities (Table 1). However, fundoscopy revealed retinal arterial spasm and hemorrhage (Figure 1). Based on the patient’s clinical symptoms and auxiliary test results, a diagnosis of severe preeclampsia was established. Magnesium sulfate was administered for anticonvulsant therapy, labetalol for blood pressure reduction, and diazepam for sedation. Owing to the severity of her condition, a cesarean section under epidural anesthesia was performed to terminate the pregnancy, which resulted in the delivery of a female infant weighing 3,550 g, with an Apgar score of 9–9. During surgery, a total of 1,100 mL of fluid was infused, with an estimated blood loss of 300 mL, and a urine output of 50 mL. Postoperatively, anticonvulsant therapy, blood pressure management, and fluid infusion (2,200 mL) were continued. However, on the night of the surgery, the patient’s blood pressure remained elevated, fluctuating between 134–175/93–126 mmHg. On the first postoperative day, her oxygen saturation dropped to 90–91%. After the administration of oxygen via a nasal cannula, her oxygen saturation improved to 97%. Further testing revealed significant abdominal fluid accumulation, diffuse pulmonary exudates, and a small pleural effusion (Figure 2). Laboratory results showed a hemoglobin level of 154 g/L, hematocrit of 45.6%, albumin of 23.1 g/L, creatinine of 131.9 μmol/L, and a significantly elevated D-dimer level of 18,810 μg/L. After an abdominal puncture and drainage of approximately 500 mL of clear fluid, 10 g of albumin was administered intravenously. Furthermore, 4,000 units of low-molecular-weight heparin were administered subcutaneously to prevent thrombosis. Blood pressure management, anticonvulsant therapy, and other symptomatic treatments were continued. Despite these interventions, the patient displayed limited improvement, experiencing somnolence as well as a decrease in urine output (0.3 mL/kg·h) and hypotension, with a blood pressure of 103/51 mmHg. Head computed tomography revealed no abnormalities, and repeat arterial blood gas analysis showed the following: pH 7.37, partial pressure of oxygen 78.00 mmHg, lactate 1.20 mmol/L, bicarbonate 20.7 mmol/L, and oxygenation index of 226. Based on the patient’s clinical manifestations and diagnostic findings (including drowsiness, hypoxia, blood gas abnormalities, hypotension, oliguria, massive abdominal ascites, pulmonary exudation, blood concentration, hypoalbuminemia, and elevated blood creatinine), after ruling out differential diagnoses such as HELLP syndrome, sepsis, and pulmonary embolism, the patient was diagnosed with CLS. Following continued intravenous administration of 10 g albumin, the patient’s urine output gradually increased, with a 24-h urine volume of 3,600 mL, a negative fluid balance of 860 mL, improved consciousness, and blood pressure fluctuations between 122–145/81–97 mmHg. On the second postoperative day, hydroxyethyl starch (30 g BID) and dexamethasone (5 mg) were incorporated into the treatment regimen to increase plasma osmolality and improve endothelial function. In addition, albumin and low-molecular-weight heparin were administered to prevent thrombosis. The patient’s urine output rapidly increased to 6,600 mL, resulting in a negative fluid balance of 4,100 mL. Chest and abdominal effusions decreased; meanwhile, oxygen saturation improved to 92–93%. Additionally, the laboratory parameters demonstrated significant improvement (Table 2). By the third postoperative day, after the discontinuation of oxygen therapy, oxygen saturation was maintained at 94%, and blood pressure stabilized at 118–137/80–91 mmHg. The 24-h urine output was 5,500 mL, with a negative fluid balance of 2,700 mL. On the fourth and fifth postoperative days, the patient’s condition continued to improve. The abdominal drainage tube was removed on the fifth day. The patient was discharged in good condition. At the 6-month follow-up, no recurrence of similar symptoms was noted, and the patient was in good health.

**Table 1:** Admission assessment.

Category	Details
Chief complaints	Amenorrhea for 37 + weeks, bilateral lower limb edema for half a month.
Vital signs	Temperature: 36.2°C, pulse: 112 bpm, respiration: 19/min, blood pressure: 165/118 mmHg, BMI: 32 kg/m^2^, height: 165 cm, weight: 88 kg. Mild edema in the lower extremities.
Obstetric findings	Fundal height: 36 cm, Abdominal circumference: 108 cm, LOA fetal position, head presentation, fetal heart rate: 142 bpm, No uterine contractions, normal birth canal, no cervical dilation. Estimated fetal weight: 3,200 g.
Laboratory and imaging data	24-h ambulatory blood pressure (BP) monitoring 24-h average BP: 161/111 mmHg, Daytime average BP: 160/111 mmHg, Nighttime average BP: 163/109 mmHg, Fetal ultrasound: Fetal size consistent with gestational age, no abnormalities in the placenta or umbilical cord. Abdominal ultrasound (liver, gallbladder, pancreas, spleen): Hepatic steatosis, hepatic cysts, splenomegaly. Cardiac ultrasound: Mild tricuspid regurgitation. Electrocardiogram (ECG): Normal. Fundoscopy: Both eyes corrected visual acuity: 5.0. Optic disk: Red with clear borders. Retina: Smooth peripheral retina with enhanced arterial reflex. Left eye: Patchy hemorrhage near major vessels, nasal to macula. Right eye: No obvious hemorrhage or exudation. Intraocular pressure: right eye 11.2 mmHg, left eye 14.1 mmHg. 24-h urinary protein: 164 mg/24 h. No abnormalities were detected in the complete blood count, coagulation function, liver and kidney function, and myocardial enzyme profile.

**Table 2:** Laboratory parameters and vital signs during hospitalization.

Parameters	Reference range	Preoperative	Day of surgery	Postoperative day 1	Postoperative day 2	Postoperative day 3	Postoperative day 4
Blood pressure (mmHg)	—	132–143/89–92	134–175/93–126	122–145/81–97	114–132/62–82	118–137/80–91	119–136/79–92
Oxygen saturation without O_2_ (%)	95	99	97	90–91	92–93	94	99
Oxygen partial pressure (mmHg)	83–108	—	—	74.6	78.7	—	152
Total input (ml)	—	—	3,300	3,340	3,570	3,550	2,650
Total output (ml)	—	—	1,650	4,200	7,700	6,250	2,410
Crystalloid solutions (ml)	—	—	2,600	1,200	500	500	200
Colloid solutions (ml)	—	—	500	100	1,100	600	0
Orally ingested solutions (ml)	—	—	200	2040	1970	2,450	2,450
Fluid balance (ml)	—	—	+1,650	−860	−4,130	−2,700	+240
Colloid osmotic pressure (mmHg)	25–28	24.5	—	17.7	18.9	19.3	23.1
Lactate level (mmol/L)	0.5–1.6	—	—	1.3	0.6	—	0.7
Hemoglobin (g/L)	115–150	131	—	157	123	109	118
Hematocrit (%)	35–45	39	—	48.1	36.7	33	36.1
C-reactive protein (mg/L)	0–10	0.29	—	46.7	37.1	25.3	—
white blood cell (10^9^/L)	3.5–9.5	6.71	-	14.43	8.97	7.02	-
Albumin (g/L)	40–55	34.2	—	23.1	25.5	26.2	32.3
Creatinine (μmol/L)	41–73	43.4	—	131.9	52.3	46.3	—
Alanine transaminase (U/L)	7–40	8.6	—	9	9.5	8.4	—
D-dimer (μg/L)	0–550	2080	—	18,810	8,100	7,890	1840
Lactate dehydrogenase (U/L)	120–250	200	—	337	—	—	—
B-type natriuretic peptide (pg/mL)	0–100	—	—	128	—	199	—

## Discussion

CLS is a clinical disorder characterized by hypoproteinemia, hypotension, hypoxemia, oliguria, and generalized edema (7). The pathophysiology of CLS is complex and often involves excessive activation of systemic inflammatory responses, which are triggered by various underlying factors. This heightened inflammatory response leads to the release of numerous inflammatory mediators, which damage the endothelial cells of the capillaries and increase their permeability. Consequently, plasma proteins and colloidal fluids leak into the extravascular compartments, resulting in reduced circulating blood volume, the development of tissue edema, and ultimately, the onset of multiorgan dysfunction (8, 9). Acute kidney injury (AKI) is the most prevalent organ dysfunction observed in CLS (10). The condition is typically caused by decreased renal perfusion secondary to a reduction in circulating blood volume. In this case, the patient exhibited hypoxemia, pleural and peritoneal effusions, hypotension, a significant decrease in plasma albumin levels on postoperative day 1, oliguria, hemoconcentration, and elevated creatinine levels. These clinical manifestations were consistent with the characteristic features of CLS.

The development of CLS in this patient after surgery may have been associated with underlying severe preeclampsia, which potentially triggered systemic small-vessel spasms and endothelial damage. However, following pregnancy termination, improvements in vascular spasm and endothelial injury were expected, potentially halting the progression of the condition. In addition, the patient’s preoperative D-dimer level was mildly elevated (2080 μg/L), indicating a mild hypercoagulable state. However, this condition is considered manageable in pregnant women at 37 weeks of gestation. The preoperative C-reactive protein (CRP) level was within the normal range (0.29 mg/L), suggesting the absence of a significant systemic inflammatory response. Surprisingly, this patient did not exhibit any clinical signs of CLS, including hypoalbuminemia, hypotension, or significant fluid accumulation, prior to surgery. Instead, a series of symptoms that were absent before and during the cesarean section emerged after pregnancy termination. What might be the cause of this? After careful review and analysis, we retrospectively hypothesized that earlier pregnancy termination could have shortened the course of severe preeclampsia, mitigated vascular endothelial spasm and injury, and consequently reduced the risk of CLS. According to the guidelines of the American College of Obstetricians and Gynecologists (ACOG) (11), termination of pregnancy should be considered for patients diagnosed with severe preeclampsia at ≥34 weeks of gestation. ACOG defines severe preeclampsia by the presence of one or more of the following manifestations: uncontrolled hypertension (>160/110 mmHg), thrombocytopenia (<100 × 10^9^/L), hepatic or renal dysfunction, pulmonary edema, severe and persistent right upper quadrant or epigastric pain (unrelieved by medication and not attributable to other diagnoses), and new-onset headache or visual disturbances (unresponsive to medical treatment and unexplained by other conditions). The ACOG also advises prompt delivery at any gestational age if maternal or fetal conditions deteriorate. In the present case, at 37 + weeks of gestation, the patient exhibited retinal artery spasm and hemorrhage, accompanied by 24-h ambulatory blood pressure exceeding 160/110 mmHg. These findings suggest that severe systemic microvascular spasms and endothelial injury lead to increased capillary permeability. Collectively, these clinical features met the ACOG diagnostic criteria for severe preeclampsia, reinforcing the need for pregnancy termination. Moreover, the patient’s postoperative blood pressure remained poorly controlled, with nocturnal readings fluctuating between 134–175/93–126 mmHg. A large volume of fluid was administered, which elevated capillary hydrostatic pressure. Additionally, studies indicate that postpartum plasma colloid osmotic pressure in women with preeclampsia can decrease to levels lower than those observed in healthy pregnant women (12), potentially triggering the onset of CLS. Furthermore, surgical trauma associated with the cesarean section may have exacerbated the patient’s condition. Research has demonstrated that stimuli such as surgery, anesthetic drugs, and changes in body position can provoke a stress response, leading to the release of inflammatory mediators such as interleukin (IL)-6. This release, in turn, increases the vascular permeability, contributing to tissue damage (13). In conclusion, as outlined in the revised Starling equation, fluid exchange in microcirculation is governed by hydrostatic pressure, colloid osmotic pressure, and the capillary filtration coefficient (14).

Early identification and diagnosis of CLS pose significant challenges in clinical practice, as its manifestations are non-specific and can easily be mistaken for other diseases. Differential diagnoses included HELLP syndrome, sepsis, and pulmonary embolism. HELLP syndrome was excluded as a diagnosis because the patient’s liver function, bilirubin and lactate dehydrogenase levels, and platelet counts were all within normal limits. Sepsis was ruled out as well, given that the patient did not exhibit fever, chills, or hypothermia before the onset of symptoms. Although CRP levels and white blood cell counts were mildly elevated in the early postoperative period, they gradually returned to normal, suggesting that these changes were caused by surgical stress rather than infection. The patient developed postoperative hypoxemia and had elevated D-dimer levels. Although pulmonary CT angiography was not performed, pulmonary embolism was considered unlikely due to the absence of symptoms such as chest pain, hemoptysis, or dyspnea. Echocardiography, electrocardiography, myocardial enzyme levels, and lower limb vascular ultrasonography revealed no abnormalities. Subsequent normalization of D-dimer levels, oxygen saturation, and arterial blood gas results further supported the exclusion of pulmonary embolism.

**Figure 3:**
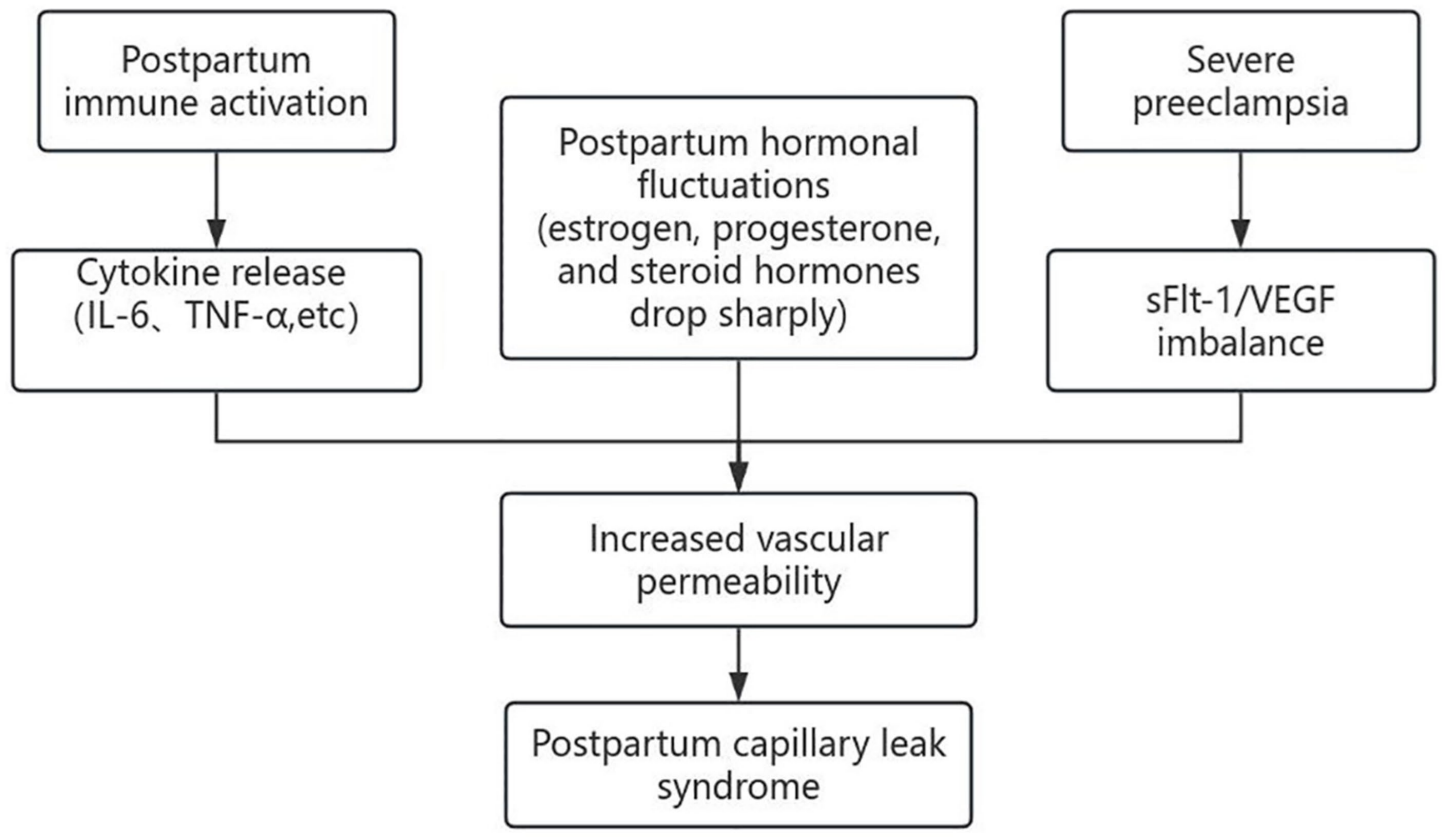
Schematic diagram illustrates the pathophysiological mechanisms of CLS induced by postpartum inflammatory and hormonal changes.

A delayed diagnosis can lead to the progression of the patient’s condition, emphasizing the critical importance of early detection. In this case, the patient’s pre-existing condition of preeclampsia further complicated the early diagnosis of CLS. The clinical manifestations of CLS closely resemble those of preeclampsia, as both conditions share common pathophysiological features, particularly involving endothelial damage. Therefore, comparing these two conditions is wise. In terms of pathogenesis, systemic small vessel spasms are a key feature of preeclampsia, while increased endothelial permeability to proteins represents the primary pathophysiological mechanism underlying CLS (3). Preeclampsia typically occurs in the late second or third trimester of pregnancy, whereas CLS is exclusively documented after childbirth (4). From a clinical perspective, severe preeclampsia may present with pathophysiological changes commonly observed in CLS, including hypoproteinemia, edema, and oliguria. Hypertension is a hallmark of preeclampsia, whereas hypotension is a distinguishing feature of CLS. Severe preeclampsia can progress to HELLP syndrome, characterized by hemolysis and thrombocytopenia, while CLS is associated with blood concentration, leading to elevated hematocrit and thrombocytosis. Typically, preeclampsia improves after pregnancy termination. However, in this case, the patient exhibited typical clinical manifestations of postpartum CLS, including hypoxemia, ascites, hypotension, oliguria, acute kidney injury, and blood concentration. The endothelial damage caused by severe preeclampsia may have increased the likelihood of developing CLS. In the presence of certain triggers, CLS is likely to develop during the postpartum period. Postpartum immune activation triggers the release of pro-inflammatory cytokines, including IL-6 and tumor necrosis factor-*α* (TNF-α), which increase capillary permeability and exacerbate vascular leakage (15). In severe preeclampsia, an imbalance in sFlt-1/vascular endothelial growth factor (VEGF) may further exacerbate vascular leakage, potentially contributing to the development of postpartum CLS (16–18). Although cytokines were not measured in this case, their role in postpartum CLS is crucial. This finding underscores the need for further studies that incorporate cytokine analysis to better understand the underlying inflammatory mechanisms; postpartum hormonal fluctuations may also play a role in CLS. During pregnancy, elevated estrogen and progesterone levels exert anti-inflammatory and endothelial stabilizing effects. However, the rapid decline in these hormones after delivery may increase susceptibility to capillary leakage (19). Similarly, a sharp decrease in steroid hormones postpartum shifts the maternal immune system from a suppressed to an activated state, which may contribute to immune imbalance and the development of CLS (20). The schematic representation is shown in Figure 3.

In the management of CLS, treatment should prioritize fluid balance, organ perfusion, and the prevention of complications. Fluid management is a critical component in the treatment of CLS (21), with the balance between intake and output adjusted progressively according to the various stages of the condition. Notably, fluid management in patients with CLS remains controversial. During the acute leakage phase, fluid supplementation should adhere to the principles of early and appropriate volume administration, with adjustments guided by central venous pressure measurements (22). Colloid solutions, particularly hydroxyethyl starch with a high molecular weight, are preferred over crystalloids. Hydroxyethyl starch not only remains in the blood vessels for a prolonged period but also reduces the release of pro-inflammatory mediators, thereby improving microcirculation (23). We believe that serum albumin levels should generally be maintained at ≥30 g/L to reduce third-space fluid accumulation and effectively preserve colloid osmotic pressure. In the recovery phase, once interstitial fluid is reabsorbed into the blood vessels, fluid supplementation should be gradually decreased. Diuretic strategies should be implemented to achieve a negative fluid balance and prevent acute pulmonary edema (24). For fluid management, in this case, we followed the principle of “goal-directed therapy,” administering fluids while closely monitoring the patient’s responsiveness. During the leakage phase, we adopted a “permissive low preload” strategy, ensuring adequate tissue perfusion while allowing for a negative fluid balance, provided that effective perfusion was maintained to support organ function. The treatment in this case proved effective, with primary fluids consisting of a combination of hydroxyethyl starch, albumin, and crystalloids, administered alongside a dexamethasone sodium phosphate injection. The patient successfully transitioned to the recovery phase 2 days post-surgery. Additionally, CLS is generally regarded as a cytokine-mediated condition, and the benefits of corticosteroids may be attributed to their ability to suppress the expression of various cytokines (25). Furthermore, during the acute leakage phase, blood becomes concentrated, which increases the risk of thrombosis, making prophylactic anticoagulation therapy essential (26). In this patient, CLS was suspected early in the postoperative period, with early diagnosis and treatment leading to favorable outcomes. A previous report documented maternal death due to postpartum CLS (27). Timely and aggressive management of the underlying conditions remains crucial. Blood pressure must be actively managed, and magnesium sulfate is used to alleviate vascular spasms and endothelial damage. Additionally, corticosteroids help improve capillary permeability. Through meticulous fluid management, continuous monitoring of fluid intake and output, and dynamic adjustments to volume load, the patient’s condition was effectively managed, preventing serious adverse outcomes.

### Patient’s perspective

If my experience can help others, I am willing to share it. When I was diagnosed with severe preeclampsia, I felt shocked and anxious. The postoperative symptoms of hypoxemia, ascites, and oliguria further heightened my worries. However, with the medical team’s prompt diagnosis and timely treatment, I gradually recovered and was saved from the dangers posed by capillary leak syndrome. I am deeply grateful to them.

## Conclusion

CLS is a rare disorder that is frequently misdiagnosed or overlooked; however, the condition progresses rapidly and is associated with a significant risk of mortality. Herein, we report a case of CLS that occurred after cesarean delivery in a patient with severe preeclampsia that was effectively diagnosed and managed. This case highlights the importance of early detection and timely intervention in the treatment of acute CLS. Patients with postpartum preeclampsia should be closely monitored to prevent fluid overload. In case of unexplained edema, hypoproteinemia, and hypovolemic shock, and after ruling out other potential conditions, CLS should be promptly considered as a possible diagnosis.

## Data Availability

The original contributions presented in the study are included in the article/supplementary material, further inquiries can be directed to the corresponding author.

## References

[1] Clarkson B Thompson D Horwith M. Cyclical edema and shock due to increased capillary permeability. Trans Assoc Am Phys. (1960) 73:272–82. doi: 10.1016/0002-9343(60)90018-8, PMID: 13693910

[2] Wollborn J Hassenzahl LO Reker D Staehle HF Omlor AM Baar W. Diagnosing capillary leak in critically ill patients: development of an innovative scoring instrument for non-invasive detection. Ann Intensive Care. (2021) 11:175. doi: 10.1186/s13613-021-00965-8, PMID: 34910264 PMC8674404

[3] Bichon A Bourenne J Gainnier M Carvelli J. Capillary leak syndrome: state of the art in 2021. Rev Med Interne. (2021) 42:789–96. doi: 10.1016/j.revmed.2021.05.012 34099313

[4] Abramov Y Galun E Granat M Barak V Abramov D Plotkin V. Postpartum systemic capillary leak syndrome: a possible etiology. Acta Obstet Gynecol Scand. (1995) 74:395–8. doi: 10.3109/00016349509024437 7778436

[5] Kanda K Sari A Nagai K Matayoshi Y. Postpartum capillary leak syndrome. A case report. Crit Care Med. (1980) 8:661–2. doi: 10.1097/00003246-198011000-00014, PMID: 7428393

[6] Eo TS Chun KJ Hong SJ Kim JY Lee IR Lee KH. Clinical presentation, management, and prognostic factors of idiopathic systemic capillary leak syndrome: a systematic review. J Allergy Clin Immunol Pract. (2018) 6:609–18. doi: 10.1016/j.jaip.2017.07.021, PMID: 28939140

[7] Siddall E Khatri M Radhakrishnan J. Capillary leak syndrome: etiologies, pathophysiology, and management. Kidney Int. (2017) 92:37–46. doi: 10.1016/j.kint.2016.11.029, PMID: 28318633

[8] Bøe OW Sveen K Børset M Druey KM. Raised serum levels of syndecan-1 (CD138) in a case of acute idiopathic systemic capillary leak syndrome (SCLS) (Clarkson’s disease). Am J Case Rep. (2018) 19:176–82. doi: 10.12659/AJCR.906514, PMID: 29449526 PMC5823032

[9] Ablooglu AJ Chen WS Xie Z Desai A Paul S Lack JB. Intrinsic endothelial hyperresponsiveness to inflammatory mediators drives acute episodes in models of Clarkson disease. J Clin Invest. (2024) 134:e169137. doi: 10.1172/JCI169137, PMID: 38502192 PMC11093607

[10] Udayabhaskaran V Arun Thomas ET Shaji B. Capillary leak syndrome following snakebite envenomation. Indian J Crit Care Med. (2017) 21:698–702. doi: 10.4103/ijccm.IJCCM_41_17, PMID: 29142382 PMC5672676

[11] Gestational Hypertension and Preeclampsia. ACOG practice bulletin, number 222. Obstet Gynecol. (2020) 135:e237–60. doi: 10.1097/AOG.0000000000003891, PMID: 32443079

[12] Bauer ST Cleary KL. Cardiopulmonary complications of pre-eclampsia. Semin Perinatol. (2009) 33:158–65. doi: 10.1053/j.semperi.2009.02.008 19464506

[13] Crippa J Mari GM Miranda A Costanzi ATM Maggioni D. Surgical stress response and enhanced recovery after laparoscopic surgery: a systematic review. Chirurgia (Bucur). (2018) 113:455–63. doi: 10.21614/chirurgia.113.4.455, PMID: 30183575

[14] Michel CC. The formulation of his hypothesis of microvascular fluid exchange and its significance after 100 years. Exp Physiol. (1997) 82:1–30. doi: 10.1113/expphysiol.1997.sp004000, PMID: 9023503

[15] Lv M Jia Y Dong J Wu S Ying H. The landscape of decidual immune cells at the maternal-fetal interface in parturition and preterm birth. Inflamm Res. (2025) 74:44. doi: 10.1007/s00011-025-02015-6, PMID: 40038160 PMC11880140

[16] Tomimatsu T Mimura K Endo M Kumasawa K Kimura T. Pathophysiology of preeclampsia: an angiogenic imbalance and long-lasting systemic vascular dysfunction. Hypertens Res. (2017) 40:305–10. doi: 10.1038/hr.2016.152, PMID: 27829661

[17] Xue S Chen J Shi Y Zhang L Chen M Sun H. Severe late onset capillary leak syndrome post Allo-HSCT successfully treated by bevacizumab: a case report. Front Med (Lausanne). (2025) 11:1483064. doi: 10.3389/fmed.2024.1483064, PMID: 39839634 PMC11746010

[18] Lesterhuis WJ Rennings AJ Leenders WP Nooteboom A Punt CJ Sweep FC. Vascular endothelial growth factor in systemic capillary leak syndrome. Am J Med. (2009) 122:e5–7. doi: 10.1016/j.amjmed.2009.01.020, PMID: 19486705

[19] Stachenfeld NS Keefe DL Palter SF. Estrogen and progesterone effects on transcapillary fluid dynamics. Am J Physiol Regul Integr Comp Physiol. (2001) 281:R1319–29. doi: 10.1152/ajpregu.2001.281.4.R1319, PMID: 11557642

[20] Mastorakos G Ilias I. Maternal hypothalamic-pituitary-adrenal axis in pregnancy and the postpartum period. Postpartum-related disorders. Ann N Y Acad Sci. (2000) 900:95–106. doi: 10.1111/j.1749-6632.2000.tb06220.x, PMID: 10818396

[21] Malbrain MLNG Marik PE Witters I Cordemans C Kirkpatrick AW Roberts DJ. Fluid overload, de-resuscitation, and outcomes in critically ill or injured patients: a systematic review with suggestions for clinical practice. Anaesth Intens Ther. (2014) 46:361–80. doi: 10.5603/AIT.2014.0060, PMID: 25432556

[22] Malbrain MLNG Malbrain G Ostermann M. Everything you need to know about deresuscitation. Intensive Care Med. (2022) 48:1781–6. doi: 10.1007/s00134-022-06761-7, PMID: 35932335 PMC9362613

[23] Qu Y Tang W Hao M Chen X. A preliminary study of influences of hydroxyethyl starch combined with ulinastatin on degree of edema in newborns with capillary leak syndrome. Am J Transl Res. (2021) 13:2626–34. PMID: 34017422 PMC8129267

[24] Correia RS Dos Santos DP Delgado M. Idiopathic systemic capillary leak syndrome: a clinical case. Cureus. (2023) 15:e50301. doi: 10.7759/cureus.50301, PMID: 38205445 PMC10776458

[25] Mier JW Vachino G Klempner MS Aronson FR Noring R Smith S. Inhibition of interleukin-2-induced tumor necrosis factor release by dexamethasone: prevention of an acquired neutrophil chemotaxis defect and differential suppression of interleukin-2-associated side effects. Blood. (1990) 76:1933–40. doi: 10.1182/blood.V76.10.1933.1933, PMID: 2242421

[26] Molteni M Pelitti V Galli M di Natale D Podda G Squizzato A. Uncommon presentation of systemic capillary leak syndrome: a case report with pulmonary embolism. Intern Emerg Med. (2024) 19:1063–6. doi: 10.1007/s11739-023-03479-2, PMID: 38041767

[27] Luke IW Rubenstein E. Fatal postpartum shock due to massive angioneurotic edema. The syndrome of transcapillary plasmapheresis. Am J Obstet Gynecol. (1962) 83:322–7. doi: 10.1016/s0002-9378(16)35839-2, PMID: 14467296

